# Eruption cysts: A series of 66 cases with clinical features

**DOI:** 10.4317/medoral.21499

**Published:** 2017-02-04

**Authors:** Emine Şen-Tunç, Hatice Açikel, Işıl Şaroğlu-Sönmez, Şule Bayrak, Nuray Tüloğlu

**Affiliations:** 1 Associate Professor. Department of Pediatric Dentistry, Faculty of Dentistry, University of Ondokuz Mayıs, Samsun, Turkey; 2 Research Assistant. Department of Pediatric Dentistry, Faculty of Dentistry, University of Ondokuz Mayıs, Samsun, Turkey; 3 Professor. Department of Pediatric Dentistry, Faculty of Dentistry, University of Adnan Menderes, Aydın, Turkey; 4 Associate Professor. Department of Pediatric Dentistry, Faculty of Dentistry, University of Eskisehir Osmangazi, Eskişehir, Turkey; 5 Assistant Professor. Department of Pediatric Dentistry, Faculty of Dentistry, University of Eskisehir Osmangazi, Eskişehir, Turkey

## Abstract

**Background:**

An eruption cyst (EC) is a benign, developmental cyst associated with a primary or permanent tooth. This paper presents 66 ECs in 53 patients who reported to 3 different centers in Turkey between 2014-2015.

**Material and Methods:**

53 patients (31 male, 22 female) with 66 ECs were diagnosed and treated over a 1-year period. The mean age of patients was 5.4 years (minimum 5 months, maximum 11 years). Clinical examination and periapical radiographs were used to establish diagnosis. Age, gender, site, history of trauma and type of treatment were recorded.

**Results:**

Of the 66 ECs diagnosed in 53 patients, more than half (56.6%) were located in the maxilla, with the maxillary first primary molars the teeth most commonly associated with ECs (30.3%). Multiple ECs were diagnosed in 13 of the 53 patients. ECs had previously diagnosed in the primary dentition of 2 patients, 3 patients reported a history of trauma to primary teeth. In the majority of patients (46 cases, 86.8%), no treatment was provided, whereas surgical treatment was provided in the remaining 7 cases (13.2%).

**Conclusions:**

Eruption cysts are usually asymptomatic and do not require treatment;. however, if the cyst is symptomatic, it should be treated with simple surgical excision.

**Key words:**Odontogenic cyst, children, eruption cyst, oral pathology.

## Introduction

An eruption cyst (EC) is a benign, developmental odontogenic cyst that accompanies an erupting primary or permanent tooth, forming shortly before the tooth’s appearance in the oral cavity (1). In the past, they were classified as a dentigerous cyst (DC) but according to the World Health Organization’s classification of odontogenic tumors, the EC is a seperate entity; it is a form of dentigerous cyst lying in the soft tissues with no bone involvement (2). There are a number of theories as to the origin of ECs, such as early caries, trauma, infection, lack of space for eruption and genetic predisposition; however, the exact etiology behind the development of an EC is unclear (3-5).

Clinically, an EC appears as a soft, translucent, dome-shaped lesion filled with blood or a clear fluid overlying the crown of an erupting tooth (6). ECs normally present during the first decade of life in either singular or multiple, unilateral or bilateral form (7).

There are numerous studies in the dental literature on ECs (1,5,6,8-12), most of which report on individual cases.So there is a lack of common consensus about their diagnosis and treatment procedure. This study reports on 66 EC cases that presented at 3 different centers in different regions in Turkey over a one-year period and includes data on the age, gender, site, history of trauma and type of treatment for each case.

## Material and Methods

This study was conducted with 53 patients ranging in age from 5 months to 11 years old who were referred to 1 of 3 different dental centers in Turkey for the management of an EC. The study was approved by the human ethics committee of Ondokuz Mayıs University (No:2016/212-14), and all procedures were conducted in accordance with the 1975 Helsinki Declaration (as revised in 2008). Diagnosis was established based on clinical examination and, if the child showed adequate cooperation, periapical radiographs. Patients with a superficial swelling located over the crown of a tooth in the eruption stage shortly before the emergence of the tooth into the oral cavity were included in the study if radiographic characteristics showed no bone involvement. Swellings varied in size and color from transparent to blue, purple and blue-black (3,7). For each EC, patient age, gender, presentation site, type of treatment provided and possible etiological factors (trauma, genetic predisposition) were recorded.

Initial treatment of each EC was based on massage on the area of the lesion and close monitoring of the lesion for allowing spontaneous regression unless infection symptoms. The possibility of surgical intervention was not ruled out in the case of a lack of spontaneous regression. The treatment plan was explained to the child’s parents, and their written consent was obtained before treatment. Follow-up examinations were performed 15 days intervals. Throughout the entire course of follow-up, no treatment but only control was performed if there wasn’t any sign of discomfort or feeding difficulty. However, if the cyst showed signs of infection or the teeth did not erupt spontaneously, then surgical treatment was performed.

Descriptive statistics (mean age of participants and distribution of ECs according to dentition type, patient sex and age, and tooth type) were calculated and recorded.

## Results

In total, 53 patients with 66 ECs were included in the study. Mean age of patients was 5.4 years (age range: 5 months-11 years), and the majority of patients 31 (58.4%) were male (females: n=22, 41.6%). ECs were associated with permanent dentition in 58.5 % of the cases, the primary dentition in 41.5 %. Clinical characteristics of the cases associated with permanent and primary dentition are given in  Table 1, Table 2, respectively. The most frequently affected primary teeth were maxillary primary first molars (62.5%) and the most frequently affected permanent teeth were maxillary permanent central incisors (55.8%). Multiple ECs were observed in 13 patients (24.5%), and of these, 10 were in primary dentition. Three patients had been previously diagnosed with EC in primary dentition, and 2 patients reported a traumatic dental injury to primary teeth.

**Table 1:**
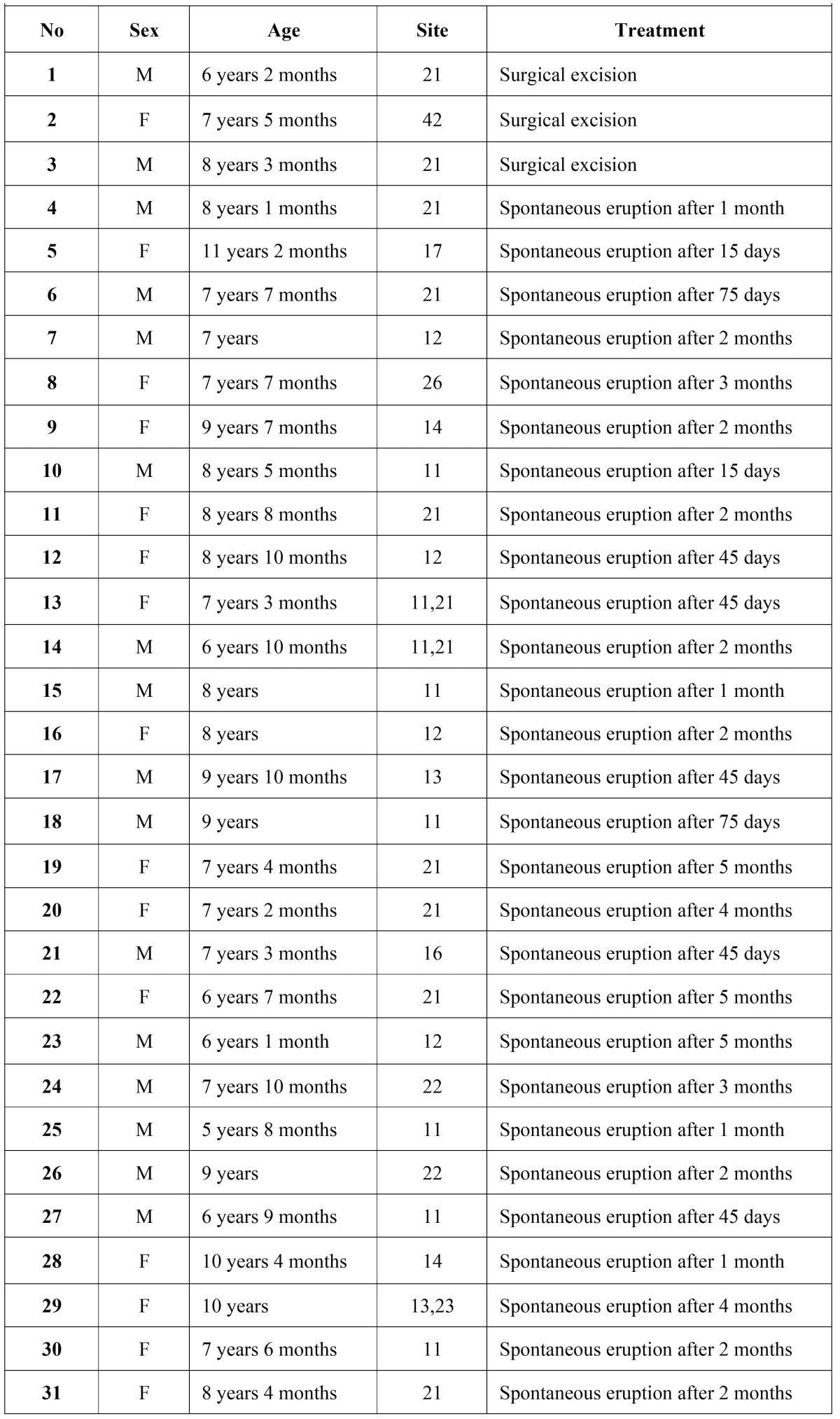
Distribution of ECs according to sex, age, site, and treatment type in permanent dentition.

**Table 2:**
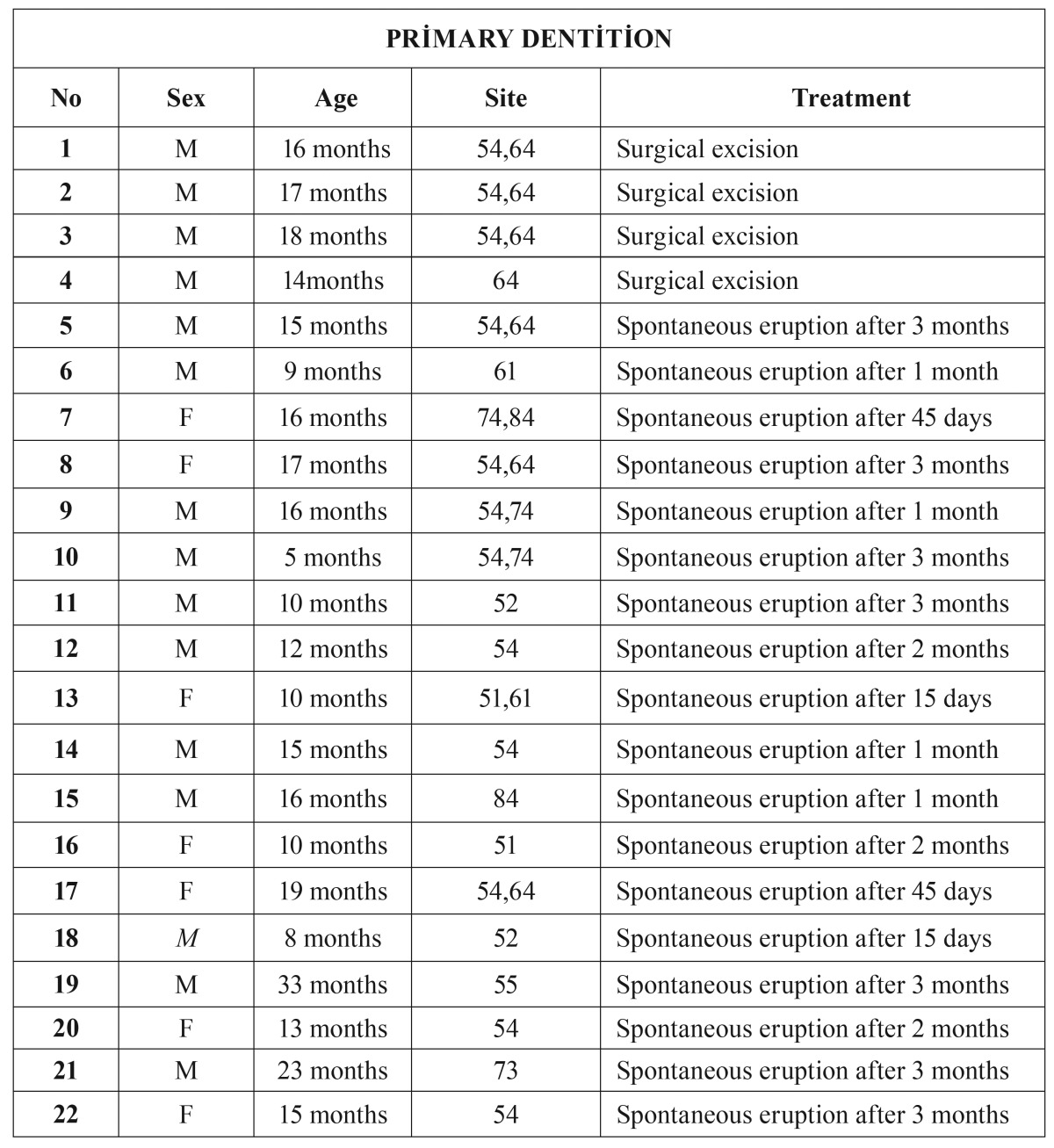
Distribution of ECs according to sex, age,site, and treatment type in primary dentition.

Clinically, most ECs presented as a raised, bluish gingival mass on the alveolar ridge. EC size varied depending upon whether the cyst was associated with a primary or permanent tooth.

The majority (n=46, 86.8%) of ECs were not associated with any discomfort and were thus initially followed-up without active intervention with the exception of recommending massaging the lesion during the observation period. The teeth associated with all these lesions erupted without any complications in approximately 2 months. However, in 7 cases (13.2%), the presence of the cyst caused a dull, aching pain on mastication, and both patients and their parents were concerned about the unaesthetic appearance. Therefore, in these cases, the ECs were treated by incising them and draining their contents. Of these 7 cases, surgical exposure was carried out under local anesthesia in 3, whereas 4 cases required sedation with nitric oxide due to the age and uncooperative nature of the patient. In all 7 cases, teeth were clinically apparent within a few weeks following surgical intervention.

## Discussion

While the process of dental eruption tends to progress without any major events, it can be a stressful experience, especially if something unusual occurs during this period. The sight of an EC, a type of lesion associated with erupting teeth, can cause panic in parents, who may jump to the conclusion that this often large, bluish-purple or bluish-black lesion is some kind of malignant tumor (13). Increasing the information available about the nature of these lesions can help clinicians to better manage these cases and relieve parents’ concerns.

The etiology of ECs remains unknown. While some authors believe degenerative cystic changes in the enamel epithelium or remaining dental lamina occurring during the process of amelogenesis play a role in the development of ECs (2,8), others have proposed early caries and chronic periapical inflammation or trauma, infection and a lack of space for eruption as possible etiological factors (3). Of the 53 patients reported on here, only 3 had had a traumatic dental injury to primary dentition, and only 2 had been previously diagnosed with EC associated with a primary tooth, whereas the rest had no known causative factor associated with their EC.

In one recently published case report, an EC developed in a patient who received cyclosporin-A (4). Another case in which drug administration was suggested in connection with EC formation was described by Nomura *et al,* who reported multiple ECs in a 4-year-old boy with Menkes Kinky Hair Disease who was treated with an anticonvulsant (diphenylhydantoin) (14). In the present study, none of the patients had taken any medication, and none had any systemic diseases.

The gender predilection of EC is controversial. Some authors have reported higher prevalence among females (3), whereas others have reported a much greater prevalence among males (2:1 male:female ratio) (15,16). The present study found a 1.4:1 male-to-female ratio for EC.

EC usually presents in the first or second decades of life (8), with the average reported age about 7 years (7). Our study found the mean age of EC cases to be 5.4 years, which is consistent with the literature, albeit slightly lower (3,15,16). The difference is most likely due to the fact that our study included many patients aged under 1 year.

Previous studies suggested that the majority of ECs are located in the incisal and molar areas, followed by the canine and premolar areas (15,16). In our study, 6 ECs were associated with deciduous maxillary incisors, 19 with deciduous maxillary molars, 31 with permanent maxillary incisors, 3 with permanent canines and 2 with permanent premolars. This distribution is in agreement with the literature.

EC is not detectable on radiographic examination because it is difficult to distinguish the cystic space of eruption cyst because both the cyst and tooth are directly in the soft tissue of the alveolar crest. Although dentigerous cysts (DCs) were considered in the differential diagnosis, DCs include a well-defined, unilocular radiolucent area, whereas no bone involvement is seen with ECs (3,7,10,17). An EC also needs to be distinguished from a hemangioma, neonatal alveolar lymphangioma, pyogenic granuloma and amalgam tattoo in differential diagnosis (10).

In most cases, EC subsides spontaneously; therefore, the recommended treatment is close monitoring with follow-up throughout the eruptive process of the involved teeth (8,11,12). When surgical treatment is decided first oral hygiene instructions are given. Generally surgical approach involves simple excision and exposure of the crown. Close proximity of the underlying tooth should not be forgotten. The treatment of drug related EC’s that complicated periodontal healing, may require scalling, root planning and special flep design (4). In the present study, conservative approach was selected in 46 cases, with massage of the lesion area recommended during the observation period. Only 7 of the 53 patients (13.2%) were treated surgically with a simple excision and exposure of the crown.
